# Selective adenosine A_2A _receptor agonists and antagonists protect against spinal cord injury through peripheral and central effects

**DOI:** 10.1186/1742-2094-8-31

**Published:** 2011-04-12

**Authors:** Irene Paterniti, Alessia Melani, Sara Cipriani, Francesca Corti, Tommaso Mello, Emanuela Mazzon, Emanuela Esposito, Placido Bramanti, Salvatore Cuzzocrea, Felicita Pedata

**Affiliations:** 1 Department of Clinical and Experimental Medicine and Pharmacology, School of Medicine, University of Messina, Italy; 2 Department of Pharmacology, University of Florence, Italy; 3 Department of Clinical Pathophysiology, Gastroenterology Unit, University of Florence, Italy; 4 IRCCS Centro Neurolesi "Bonino-Pulejo", Messina, Italy

## Abstract

**Background:**

Permanent functional deficits following spinal cord injury (SCI) arise both from mechanical injury and from secondary tissue reactions involving inflammation. Enhanced release of adenosine and glutamate soon after SCI represents a component in the sequelae that may be responsible for resulting functional deficits. The role of adenosine A_2A _receptor in central ischemia/trauma is still to be elucidated. In our previous studies we have demonstrated that the adenosine A_2A _receptor-selective agonist CGS21680, systemically administered after SCI, protects from tissue damage, locomotor dysfunction and different inflammatory readouts. In this work we studied the effect of the adenosine A_2A _receptor antagonist SCH58261, systemically administered after SCI, on the same parameters. We investigated the hypothesis that the main action mechanism of agonists and antagonists is at peripheral or central sites.

**Methods:**

Spinal trauma was induced by extradural compression of SC exposed via a four-level T5-T8 laminectomy in mouse. Three drug-dosing protocols were utilized: a short-term systemic administration by intraperitoneal injection, a chronic administration via osmotic minipump, and direct injection into the spinal cord.

**Results:**

SCH58261, systemically administered (0.01 mg/kg intraperitoneal. 1, 6 and 10 hours after SCI), reduced demyelination and levels of TNF-α, Fas-L, PAR, Bax expression and activation of JNK mitogen-activated protein kinase (MAPK) 24 hours after SCI. Chronic SCH58261 administration, by mini-osmotic pump delivery for 10 days, improved the neurological deficit up to 10 days after SCI. Adenosine A_2A _receptors are physiologically expressed in the spinal cord by astrocytes, microglia and oligodendrocytes. Soon after SCI (24 hours), these receptors showed enhanced expression in neurons. Both the A_2A _agonist and antagonist, administered intraperitoneally, reduced expression of the A_2A _receptor, ruling out the possibility that the neuroprotective effects of the A_2A _agonist are due to A_2A _receptor desensitization. When the A_2A _antagonist and agonist were centrally injected into injured SC, only SCH58261 appeared neuroprotective, while CGS21680 was ineffective.

**Conclusions:**

Our results indicate that the A_2A _antagonist protects against SCI by acting on centrally located A_2A _receptors. It is likely that blockade of A_2A _receptors reduces excitotoxicity. In contrast, neuroprotection afforded by the A_2A _agonist may be primarily due to peripheral effects.

## Background

Spinal cord injury (SCI) is a devastating and complex clinical condition that produces a predictable pattern of progressive injury entailing neuronal loss, axonal destruction and demyelination at the site of impact [1]. Ultimately, neuronal deficits/dysfunction result. Although innovative medical care has improved patient outcome, advances in pharmacotherapy to limit neuronal deficits and promote regeneration and function have been limited. Primary traumatic mechanical injury to spinal cord (SC) causes death of neurons that cannot be recovered and regenerated. Studies have indicated that neurons continue to die for hours following traumatic SCI [2] and that demyelination occurs [3]. Normally acute injury leads to chronic injury in the SC. The events that characterize this successive phase of mechanical injury are called "secondary damage." It is now accepted that a local inflammatory response amplifies the secondary damage. Evidence indicates that resident microglia and macrophages originating from blood are two key cell types related to the occurrence of neuronal degeneration in CNS after traumatic injury. In particular, when SCI occurs, microglia in parenchyma are activated and macrophages from the circulation are able to cross the blood-brain barrier to act as intrinsic spinal phagocytes [4,5].

Currently, drugs used to treat acute spinal cord injury attempt to prevent secondary inflammatory neuronal damage [6]. Accordingly, several studies have shown that therapies targeting various factors involved in the secondary degeneration cascade lead to tissue sparing and improved behavioral outcomes in spinal cord-injured animals [7-10]. Among different therapies, several studies have demonstrated that adenosine A_2A _receptor agonists protect against locomotor dysfunction following SC ischemia-reperfusion and traumatic injury [11-15]. We have previously demonstrated that, 24 hours after SC trauma, A_2A _receptor agonists reduce influx of MPO-positive leukocytes, NF-kB activation and iNOS expression in traumatized tissue [14], as well as expression of death signals such as tumor necrosis factor-α (TNF-α), caspase-3, Fas-L, annexin-V, and BAX, while Bcl-2 expression is increased [15]. In addition to reduction of inflammatory and apoptotic pathways, A_2A _agonists reduce activation of JNK mitogen-activated protein kinase (MAPK) in oligodendrocytes 24 hours after SCI [14]. Since JNK MAPK activation contributes to activation of caspase-3 and of the proapoptotic regulator DP5 in oligodendrocytes and neurons of injured SC following traumatic spinal cord injury [16], reduction of JNK MAPK activation might account for A_2A _agonist-induced protection from demyelination and neuron recovery after SCI.

Despite the definite protection afforded by A_2A _agonists in SCI, currently available information regarding the role of adenosine A_2A _receptors in central ischemia/trauma is conflicting [17]. While most studies demonstrate a protective effect of A_2A _agonists after trauma/ischemia in SC, robust evidence from studies of brain indicates that A_2A _receptor genetic inactivation [18] and adenosine A_2A _antagonists protect against ischemia [19-22].

Li and coworkers [13] have demonstrated that, when peripherally administered, both A_2A _agonist and antagonist are protective against locomotor dysfunction and demyelination after SCI. After lumbar laminectomy, adenosine increases extracellularly soon after trauma up to μM values [23] that are able to stimulate the four G protein-coupled receptors: A_1_, A_2A_, A_2B_, and A_3 _[24].

To shed light on the mechanism of protection of adenosine A_2A _receptor agonists/antagonists, in this study we investigated the effects of the selective adenosine A_2A _receptor antagonist, SCH58261, systemically and repeatedly administered after SCI, on inflammation parameters and on JNK MAPK activation. Moreover, we studied if adenosine A_2A _receptors display plastic changes after repeated systemic treatment with the A_2A_-selective receptor agonist CGS21680 or with the A_2A_-selective antagonist SCH58261. Finally, we examined the protective effect afforded by A_2A _agonist and antagonist after direct injection into injured SC to discern the site of action.

## Methods

### Animals

Male adult CD1 mice (25-30 g, Harlan Nossan, Milan, Italy) were housed in a controlled environment and provided with standard rodent chow and water. All experiments were carried out according to the ECC guidelines for animal care (DL 116/92, application of the European Communities Council Directive 86/609/EEC). All efforts were made to minimize animal suffering and the number of animals used.

### Spinal cord injury (SCI)

Mice were anesthetized using chloral hydrate (400 mg/kg i.p.; Sigma-Aldrich, St. Louis, MO, USA). We used the clip compression model described by Rivlin and Tator [25] and produced SCI by extradural compression of a section of the SC exposed via a four-level T5-T8 laminectomy, in which the prominent spinal process of T-5 was used as a surgical guide. A four-level laminectomy was chosen to expedite timely harvest and to obtain enough SC tissue for biochemical examination. With the aneurysm clip applicator oriented in the bilateral direction, an aneurysm clip with a closing force of 24 g was applied extradurally at T5-T8 level (for approximately 60 sec). The clip was then rapidly released with the clip applicator, which caused SC compression. In the injured groups, the cord was compressed for 1 min. Following surgery, 1.0 cc of saline was administered subcutaneously in order to replace the blood volume lost during the surgery. During recovery from anesthesia, the mice were placed on a warm heating pad and covered with a warm towel. The mice were singly housed in a temperature-controlled room at 27°C for a survival period of 20 days. Food and water were provided to the mice *ad libitum*. During this time period, the animals' bladders were manually voided twice a day until the mice were able to regain normal bladder function. Sham-injured animals were subjected only to laminectomy.

### Experimental groups

In the experiments in which SCH58261 or CGS21680 were systemically injected, mice were randomly allocated into the following groups: (i) *SCI+vehicle group*. Mice were subjected to SCI plus administration of saline 10% DMSO with an i.p. bolus (*N *= 20); (ii) *CGS21680 group*. Same as the *SCI+vehicle group *but in which CGS21680, at the dose of 0.1 mg/kg (i.p.), was administered three times: 1 h, 6 h and 10 h after SCI (*N *= 20); (iii) *SCH58261 group*. Same as the *SCI+vehicle group *but in which SCH58261, at the dose of 0.01 mg/kg (i.p.), was administered three times: 1 h, 6 h and 10 h after SCI (*N *= 20); (iv) *Sham+vehicle group*. Mice were subjected to the same surgical procedures as the above groups except that the aneurysm clip was not applied and they were treated i.p. with vehicle (saline 10% DMSO) (*N *= 20).

In the experiments in which SCH58261 or CGS21680 were centrally applied on SC tissue at 1 h, 6 h and 10 h after SCI, the applied doses were, respectively, 0.01 nmoles and 0.5 nmoles. This was determined on the basis of doses administered in microdialysis studies [26,27]. The doses of SCH58261 and CGS21680, systemically administered, were chosen on the basis of our previous *in vivo *studies [14,15,20-22].

### Mini-osmotic pump implantation and SCH58261 delivery

In the mouse group subjected to motor function evaluation, Alzet pumps were used to deliver vehicle (saline 10% DMSO) (N = 10) or SCH58261 (N = 10). SCH58261 (0.01 mg/kg) was delivered at a constant rate for 10 days after injury. In particular, we used Alzet Model 2002 mini-osmotic pumps (Charles River Milan Italy), placed 3 hours after SCI. The Alzet mini-osmotic pump was implanted subcutaneously (s.c.) in the mouse, as previously described by Genovese et al. [14,15]. A small incision was made in the skin between the scapulae. Using a hemostat, a small pocket was formed by spreading the subcutaneous connective tissues apart. The pump was inserted into the pocket with the flow moderator pointing away from the incision. The skin incision was closed with suture clips (Aesculap Surgical Instruments). The pumping rate was 0.5 μl/h (± 0.15 μl/h) and the reservoir volume was 200 μl.

### Grading of motor disturbance and light microscopy

Locomotor performance of animals was analyzed using the Basso mouse scale (BMS) open-field score [28] 10 day after injury, since the BMS has been shown to be a valid locomotor rating scale for mice. The evaluations were made by two observers blinded to all analyzed groups. Briefly, the BMS is a nine-point scale that provides a gross indication of locomotor ability and determines the phases of locomotor recovery and features of locomotion. BMS scale ranges from 0 (indicating complete paralysis) to 9 (indicating normal hindlimb function), and are based on rating locomotion on aspects of hindlimb function such as weight support, stepping ability, coordination, and toe clearance. The BMS score was determined for ten mice in each group.

Twenty-four hours following trauma, the animals were anaesthetized with chloral hydrate (400 mg/kg i.p.) and sacrificed by decapitation, and spinal cord tissues were dissected. Tissue segments containing the lesion (1 cm on each side of the lesion, T5-T8) were paraffin embedded and cut into longitudinal 5-μm-thick sections for the posterior area of the spinal cord. Tissue sections (thickness 5 μm) were deparaffinized with xylene, stained with hematoxylin/eosin, Luxol fast blue Kluver Barrera for myelin, Weigert's iron hematoxylin for nuclei and Oil red O for lipids, and studied using light microscopy (Dialux 22 Leitz).

Segments of each SC were evaluated by an experienced histopathologist. Damaged neurons were counted and the histopathologic changes in gray matter were scored on a 6-point scale: 0, no lesion observed, 1, gray matter contained 1 to 5 eosinophilic neurons; 2, gray matter contained 5 to 10 eosinophilic neurons; 3, gray matter contained more than 10 eosinophilic neurons; 4, small infarction (less than one-third of the gray matter area); 5, moderate infarction; (one-third to one-half of the gray matter area); and 6, large infarction (more than half of the gray matter area). Scores from all sections from each SC were averaged to give a final score for each individual mouse. All the histological studies were performed in a blinded fashion.

### Immunohistochemical localization of TNF-α, PAR, Bax and Bcl-2, Fas Ligand

Twenty-four hours after SCI, tissues were fixed in 10% (w/v) paraformaldehyde. After deparaffinization, endogenous peroxidase was quenched with 0.3% (v/v) hydrogen peroxide in 60% (v/v) methanol for 30 min. The sections were permeabilized with 0.1% (w/v) Triton X-100 (TX) in phosphate buffer solution (PBS) for 20 min. Non-specific adsorption was minimized by incubating the sections in 2% (v/v) normal goat serum in PBS for 20 min. Endogenous biotin or avidin binding sites were blocked by sequential incubation for 15 min with biotin and avidin (DBA), respectively. Sections were incubated overnight with anti-TNF-α (Santa Cruz Biotechnology; 1:500 in PBS, v/v), anti-PAR antibody (1:500 in PBS, v/v), anti-FAS-ligand antibody (Abcam,1:500 in PBS, v/v), anti-Bax antibody (Santa Cruz Biotechnology, 1:500 in PBS, v/v) or anti-Bcl-2 polyclonal antibody (Santa Cruz Biotechnology, 1:500 in PBS, v/v). Sections were washed with PBS and incubated with secondary antibody. Specific labeling was detected with a biotin-conjugated goat anti-rabbit IgG and avidin-biotin peroxidase complex (DBA). To verify the binding specificity for TNF-α, FAS-L, PAR, Bax, and Bcl-2, some sections were also incubated with only the primary antibody (no secondary) or with only the secondary antibody (no primary). In these situations no positive staining was found in the sections indicating the specificity of the positive immunoreactions in all the experiments carried out.

### Fluorescence deconvolution microscopy

Twenty-four hours after SCI, mice were transcardically perfused, under deep anesthesia, with ice-cold 4% paraformaldehyde solution (in phosphate buffer, pH 7.4). Spinal cords were post-fixed overnight and cryoprotected in an 18% sucrose solution (in phosphate buffer) for at least 48 h. Spinal cords were cut with a cryostat and 30 μm-thick coronal sections were collected. Sections were placed in antifreeze solution (30% ethylene glycol, 30% glycerol in phosphate buffer) and stored at -20°C until assay.

The cellular types that expressed A_2A _receptor were identified, using fluorescence microscopy, in 30 μm-thick coronal sections cut and stored as described above.

#### Day 1

Free-floating sections were washed in PBS-TX for 10 min, then incubated at room temperature in blocking buffer for 40 min. Sections were then incubated, overnight at room temperature, with a mouse monoclonal primary antibody against the A_2A _receptor (1:400 anti-A_2A _receptor, Millipore) and a rabbit polyclonal antibody anti-glial fibrillary acid protein (GFAP, 1:500; Abcam) used to visualize astrocytes, or with rabbit polyclonal antibody IBA1 (1:300; Wako) used to visualize microglia, or stained with NeuroTrace green fluorescent Nissl stain (Nissl, 1:200; Invitrogen) used to visualize neurons, or immunoreacted with a rabbit polyclonal antibody anti-oligodendrocyte specific protein (OSP, 1:100; Abcam) used to visualize oligodendrocytes. OSP is described in the white matter tracts of rat spinal cord, predominantly in laminar myelin [29].

#### Day 2

After washing in PBS-TX (3 times, 10 min each), slices were incubated for 2 h at room temperature in the dark with Texas red-conjugated goat anti-mouse IgG (1:400 Vectastain, Vector Laboratories, Burlingame, CA, USA) and fluorescein-(FITC)-conjugated goat anti-rabbit IgG (1:400) in blocking buffer. After extensive washings, slices were mounted using Vectashield (Vectastain, Vector Laboratories, Burlingame, CA, USA) as a mounting medium.

Images were collected through a 40 × 0.75 NA objective on a Leica DM6000B microscope equipped with a DFC350FX B/W camera. Each sample was acquired as a Z-stack (in 0.74 um steps) and deconvolved with Huygens Professional software (SVI, Netherlands). Deconvolution was performed using the CLME algorithm and the theoretical PSF. Images are presented as maximum intensity projection (Image J software) of the whole z-stacks acquired. The images were then assembled into montages using Adobe Photoshop 7.0 (Adobe Systems, Mountain View, CA, USA).

To verify the binding specificity of anti-A_2A _receptor, GFAP, IBA1, OSP, Nissl antibodies some sections were incubated with only the secondary antibody (no primary). In these situations no positive staining was found.

### Western blot for A_2A _receptor and phospho JNK MAPK

Briefly, SC tissues from each mouse were suspended in extraction buffer A containing 0.2 mM PMSF, 0.15 μM pepstatin A, 20 μM leupeptin, 1 mM sodium orthovanadate; homogenized at the highest setting for 2 min, and centrifuged at 1,000 × g for 10 min at 4°C. Supernatants represented the cytosolic fraction. The pellets, containing enriched nuclei, were re-suspended in Buffer B containing 1% Triton X-100, 150 mM NaCl, 10 mM TRIS-HCl pH 7.4, 1 mM EGTA, 1 mM EDTA, 0.2 mM PMSF, 20 μm leupeptin, 0.2 mM sodium orthovanadate. After centrifugation for 30 min at 15,000 × g at 4°C, the supernatants containing the nuclear protein were stored at -80°C for further analysis. The level of A_2A _receptors and phospho-JNKs MAPK were quantified in cytosolic fraction from spinal cord tissue collected 24 hours after SCI. The filters were blocked with 1× PBS, 5% (w/v) non fat dried milk (PM) for 40 min at room temperature and subsequently probed with a specific Abs A_2A _receptor (Enzo Life Science, 1:200), or anti-phospho-JNK MAPK (Thr183/Tyr185) (1:1000; Cell Signaling) in 1× PBS, 5% w/v non fat dried milk, 0.1% Tween-20 (PMT) at 4°C, overnight. Membranes were incubated with peroxidase-conjugated bovine anti-mouse IgG secondary antibody or peroxidase-conjugated goat anti-rabbit IgG (1:2000, Jackson ImmunoResearch, West Grove, PA) for 1 h at room temperature.

To ascertain that blots were loaded with equal amounts of proteic lysates, they were also incubated in the presence of the antibody against GAPDH protein (1:5000 Sigma-Aldrich) or antibody against β-actin protein (1:10,000 Sigma-Aldrich). Semi-quantitative densitometric analysis of the relative expressions of the protein bands of A_2A _receptor and phospho-JNK MAPK (54 and 46 kDa) was quantified by scanning of the X-ray films with a GS-700 Imaging Densitometer (GS-700, Bio-Rad Laboratories, Milan, Italy) and a computer program (Molecular Analyst, IBM), and standardized for GAPDH or β-actin levels.

### Statistical analysis

The results were analyzed by one-way ANOVA followed by a Bonferroni post-hoc test. A p < 0.05 was considered significant.

## Results

### Systemic treatment with SCH58261 ameliorates motor function and tissue damage after SCI

**Figure 1:**
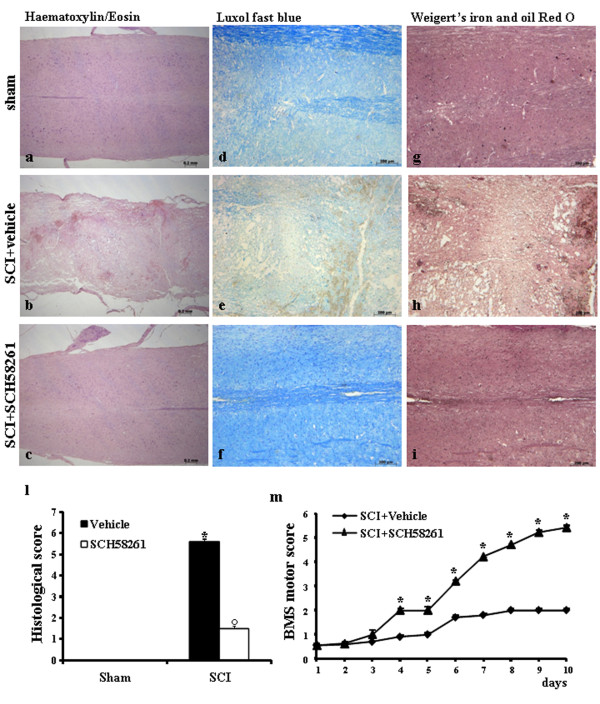
**Effects of systemic SCH58261 treatment on histological alterations and on hind limb motor disturbance after SCI**. Twenty-four hours after trauma, significant damage to the SC of untreated SCI-operated mice in the perilesional area was assessed by the presence of alterations of white matter (b). It is noteworthy that significant protection from the SCI was observed in tissue collected from SCH58261-treated SCI-injured mice (c). No significant alterations were observed in sections obtained in sham groups (a). Myelin structure was observed by Luxol fast blue staining as well by Weigert's and Oil red O staining. Twenty-four hours after injury in SCI-operated mice (e, h respectively) a significant loss of myelin was observed. In contrast, myelin degradation was attenuated (f, i respectively) in SCH58261-treated mice. No significant alterations were observed in sections obtained in sham groups (d, g respectively). This figure is representative of at least 3 experiments performed on different experimental days. The histological score (l) was evaluated by an independent observer. ND: not detectable. One-way ANOVA: *°P < 0.01 vs sham group and SCI+vehicle, respectively. The degree of motor disturbance was assessed every day until 10 days after SCI by BMS motor score (m). Systemic administration of SCH58261 reduced the motor disturbance starting from the fourth day after SCI (m). Values are shown as mean ± S.E., with 10 mice in each group. One-way ANOVA: *P < 0.01 vs SCI+vehicle.

The severity of trauma in the perilesional area was assessed by hematoxylin-eosin staining (Figure 1a-c) as well as assessment of alterations of white matter by Luxol fast blue staining (Figure 1d-f) and by Weigert's and Oil red O staining (Figure 1g-i), in SCI+vehicle-treated, SCI+SCH58261-treated and sham-operated mice group 24 h after injury. Significant damage was observed in SC tissue from SCI mice (Figure 1b) when compared with sham-operated mice (Figure 1a). The histological scores of damage were significantly reduced in SCH58261-treated mice (Figure 1c) in comparison to vehicle-treated mice (Figure 1l). In sham animals, myelin structure was clearly stained by Luxol fast blue in both lateral and dorsal funiculi of the SC (Figure 1d). At 24 h after the injury, in SCI-operated mice, a significant loss of myelin in lateral and dorsal funiculi was observed by Luxol fast blue (Figure 1e) and by Weigert's and Oil red O coloration (Figure 1h). In contrast, myelin damage was attenuated in the central part of lateral (Figure 1f) and dorsal funiculi in SCH58261-treated mice (Figure 1i).

To evaluate whether histological damage to the SC was associated with a loss of motor function, the BMS open-field score was used [28]. Motor function was not impaired in sham mice (data not shown). Mice subjected to SCI showed significant deficits in hind limb movement (Figure 1m) starting with the first evaluation performed 24 h after trauma. In chronic SCH58261-treated mice group, the neurological deficit improved in a statistically significant way beginning at four days after chronic administration, compared to the SCI+vehicle mice group, and persisting up to 10 days after SCI.

### Systemic treatment with SCH58261 protects from inflammatory parameters

Immuno-histological analysis of TNF-α, Fas-L, PAR, BAX and Bcl2 was performed to ascertain whether SCH58261 treatments modulate levels of these molecular signals that may be implicated in inflammatory response.

**Figure 2:**
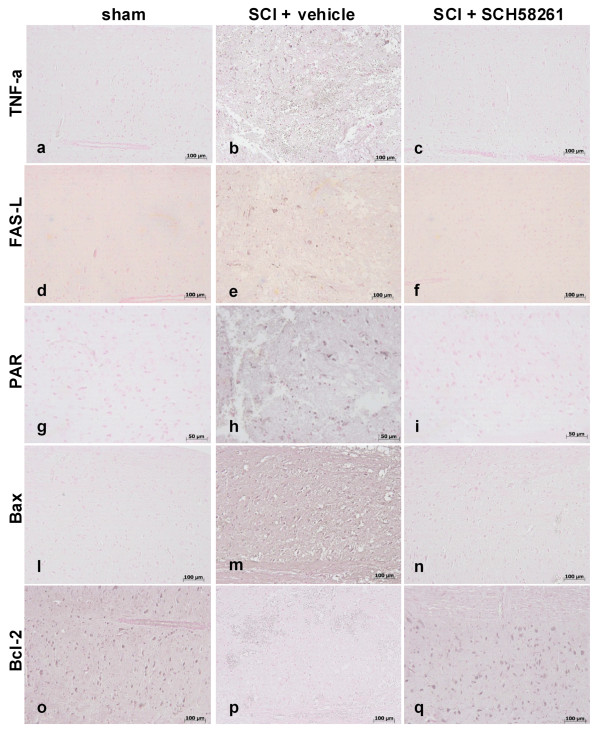
**Effects of systemic SCH58261-administration on inflammation parameters**. A substantial increase in TNF-α (2b), Fas-L (2e) and PAR (2h) expression was found in SC tissue collected from SCI+vehicle-treated mice 24 hours after SCI, in comparison with sham-operated mice (2a, d, g respectively). TNF-α (2c), Fas-L (2f) and PAR (2i) levels were attenuated in the SCI+SCH58261 group in comparison to SCI+vehicle animals. Sections of SC from sham vehicle-treated mice did not stain for Bax (2l) whereas SC sections obtained from SCI mice exhibited positive staining (2m). SCH58261 reduced the degree of staining for Bax (2n). Spinal cord sections from sham vehicle-treated mice demonstrated Bcl-2 positive staining (2o), whereas in SCI control mice the staining was significantly reduced (2p). SCH58261 attenuated the loss of positive staining for Bcl-2 in the SC from SCI-subjected mice (2q).

Substantial increases in TNF-α, (Figure 2b), Fas-L (Figure 2e) and PAR (Figure 2h) expression were found in SC tissue collected from SCI+vehicle-treated mice 24 hours after SCI, in comparison with sham-operated mice (Figure 2a, d, g, respectively). In contrast, TNF-α (Figure 2c), Fas-L (Figure 2f) and PAR (Figure 2i) death signals were attenuated in the SCI+SCH58261 group in comparison to SCI+vehicle animals.

Samples of SC tissue were also analyzed 24 h after SCI to determine immuno-histological staining for Bax and Bcl-2. Sections of SC from sham vehicle-treated mice did not stain for Bax (Figure 2l) whereas SC sections obtained from SCI vehicle-treated mice were positive for Bax (Figure 2m). SCH58261 reduced the degree of positive staining for Bax in spinal cord of mice subjected to SCI (Figure 2n). Spinal cord sections from sham vehicle-treated mice demonstrated positive Bcl-2 staining (Figure 2o), whereas in SCI control mice, this staining was significantly reduced (Figure 2p). SCH58261 attenuated the loss of positive staining for Bcl-2 in spinal cord from SCI-subjected mice (Figure 2q).

### Localization of adenosine A_2A _receptors 24 h after SCI

**Figure 3:**
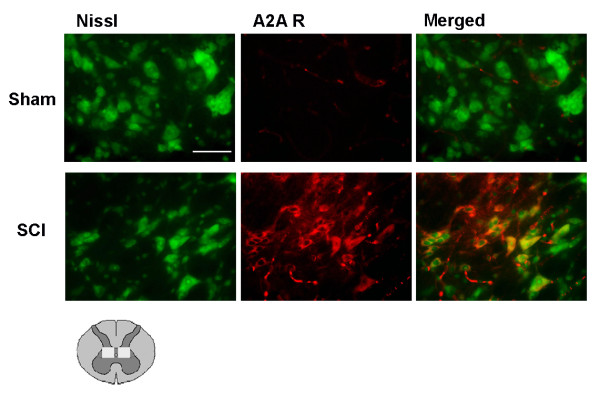
**Co-localization of adenosine A_2A _receptors with neurons (Nissl) in the gray matter of SC 24 h after injury**. Double immunofluorescence was used to characterize the co-localization of A_2A _receptors (in red with Texas red) with neurons (in green with NeutroTracer green fluorescent Nissl stain) in sham-operated and SCI mice groups. The merged images show that A_2A _receptors are present on many neurons of the gray matter after SCI, but not all neurons expressed A_2A _receptors. The drawing under the figures shows localization of A_2A _receptors as indicated by boxes. Scale bar = 50 μm.

Figure 3 shows that in sham-operated mice, only faint staining of A_2A _receptors was detectable in the gray matter of SC, indicated by the box in the drawing under the figures. Some A_2A _receptor staining was visible on blood vessels but not on Nissl-positive cells. Twenty-four hours after SCI, A_2A _receptors were definitely expressed on neurons. Costaining of A_2A _receptors with Nissl staining for neurons shows that A_2A _receptors are localized on many neurons in the central part of the gray matter while in the ventral horn of the SC, no A_2A _receptor staining was found on motoneurons.

**Figure 4:**
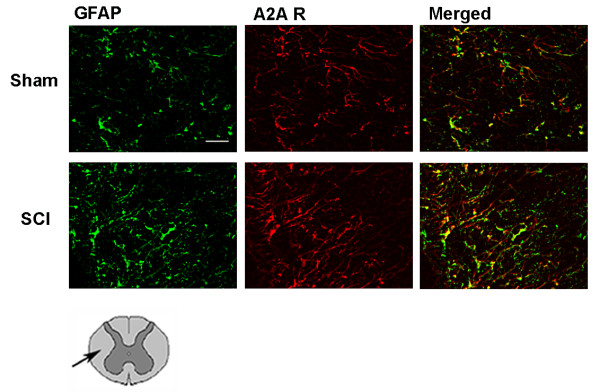
**Co-localization of A_2A _receptors with astrocytes (GFAP) in white matter of SC 24 h after injury**. Double immunofluorescence was used to characterize the co-localization of A_2A _receptors (in red with Texas red) with astrocytes (in green with fluorescein) in sham-operated and SCI mice groups. The merged images show that A_2A _receptors are present on astrocytes both before and after SCI. The co-localization is quite total. The drawing under figures shows that A_2A _receptors were identified in the white matter area as indicated by arrow. Scale bar = 10 μm.

Figure 4 shows that staining for adenosine A_2A _receptors in sham-operated mice was found also in white matter. A_2A _receptors costained with GFAP-stained cells. Twenty-four hours after SCI, astrocytes appeared fragmented with morphological features of damaged cells. In the same white matter area, 24 hours after injury, A_2A _receptor staining was slightly increased on GFAP-stained cells.

**Figure 5:**
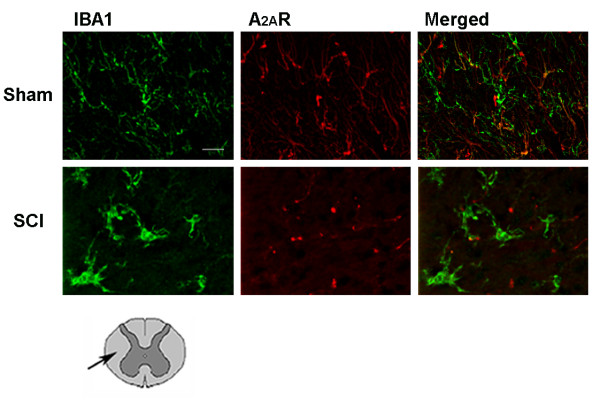
**Co-localization of A_2A _receptors with microglial cells (IBA1) in white matter of SC 24 h after injury**. Double immunofluorescence was used to characterize the co-localization of A_2A _receptors (in red with Texas red) with microglia (in green with fluorescein) in sham-operated and SCI mice groups. The merged images show that A_2A _receptors are not present on activated microglial cells of the SC. The drawing under figures shows that A_2A _receptors were identified in the white matter area as indicated by arrow. Scale bar = 10 μm.

Figure 5 shows that A_2A _receptors were expressed in only a few microglial cells in the white matter of spinal cord in sham-operated mice. Microglial cells look like a thin web with thin and long processes. Twenty-four hours after SCI, microglial cells assumed the morphological features of activated cells, with round cell body and thick and short processes. At this time after SCI, no localization of A_2A _receptors was found on activated microglia. The same pattern of microglia and A_2A _receptor colocalization was found in the gray matter.

**Figure 6:**
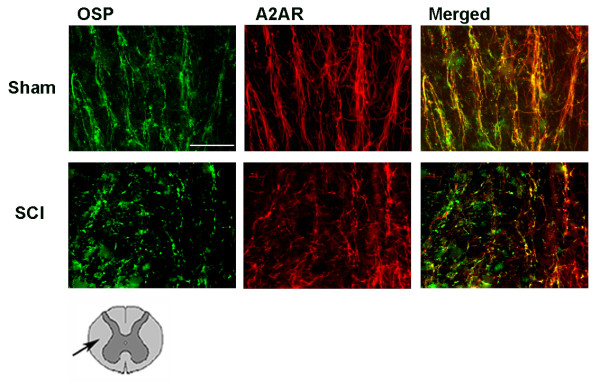
**Co-localization of A_2A _receptors with oligodendrocyte myelinated bundles (OSP) in the white matter of SC 24 h after injury**. Double immunofluorescence was used to characterize the co-localization of A_2A _receptors (in red with Texas red) with myelinated bundles (in green with fluorescein) in sham-operated and SCI mice groups. The merged images show that A_2A _receptors are present in myelinated bundles of the white matter after SCI. The drawing under figures shows that A_2A _receptors were identified in the white matter area as indicated by arrow. Scale bar = 50 μm.

Figure 6 shows the localization of A_2A _receptors on oligodendrocytes in the white matter of spinal cord 24 hours after injury. In sham-operated mice, adenosine A_2A _receptors were detectable on bundles of myelinated fibers. Twenty-four hours after SCI, bundles appeared disorganized and fragmented and there was a less definite colocalization of A_2A _receptor with oligodendrocyte processes.

### Systemic treatment with SCH58261 and CGS21680 reduces the expression of adenosine A_2A _receptors 24 h after SCI

**Figure 7:**
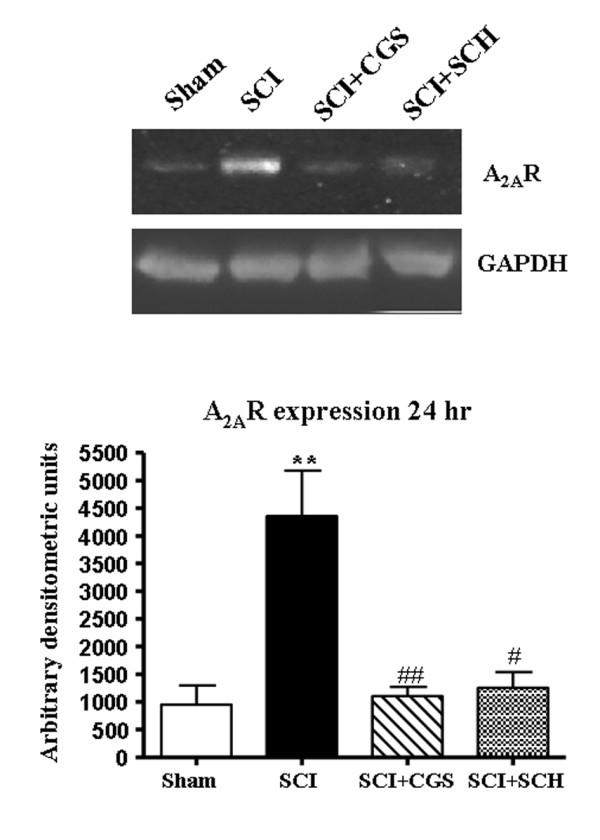
**Effects of systemic SCH58261- and CGS21680-administration on expression of A_2A _receptors**. Western blot analysis shows that A_2A _receptors are detected in the SC of sham-operated animals and that they are substantially increased in SCI mice. SCH58261 and CGS21680 treatment significantly reduced SCI-induced A_2A _receptors expression. GAPDH was used as internal control. A representative blot of lysates obtained from each mice group is shown. The densitometric analysis is expressed as the mean ± SEM for samples from 10 mice from each group and is normalized by control protein (GAPDH) levels, and reported in bar graphs. One-way ANOVA: *P < 0.01 vs sham; ^# ^P < 0.05 vs SCI; ^# # ^P < 0.01 vs SCI.

Twenty-four hours after SCI, the expression of A_2A _receptors in SC homogenates was investigated by western blot. A significant increase in A_2A _receptor levels (Figure 7) was observed in SC from mice subjected to SCI. Both SCH58261 and CGS21680 treatments prevented the SCI-induced expression of A_2A _receptor (Figure 7), when administered intraperitoneally three times in 24 hours.

### Systemic treatment with SCH58261 reduces JNK MAPK activation 24 h after SCI

**Figure 8:**
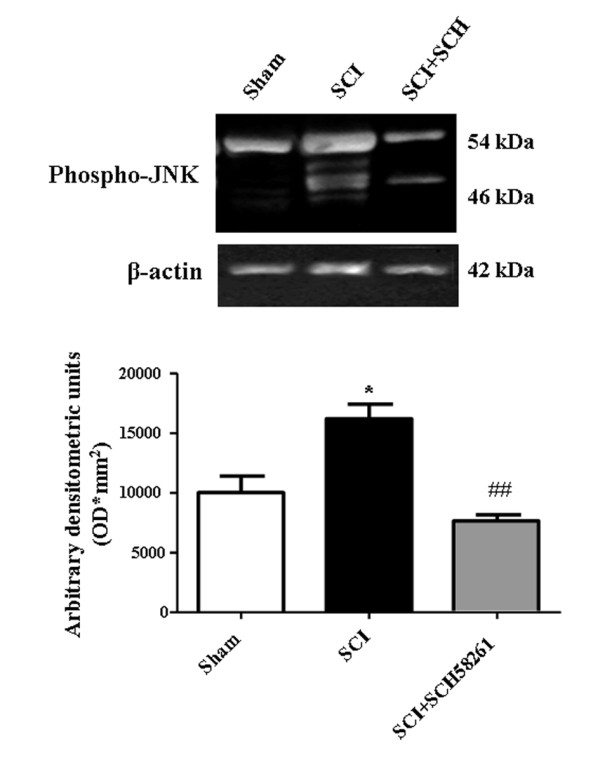
**Effect of systemic SCH58261-administration on JNK MAPK activation**. Western blot analysis shows a significant increase in phospho-JNK MAPK (Thr183/Tyr185) 24 h after SCI. SCH58261, intraperitoneally administered three times within 24 h, reduced SCI-induced phospho-JNK MAPK levels. A representative blot of lysates obtained from each mice group is shown. The densitometric analysis is expressed as the mean ± SEM for samples from 10 mice from each group and is normalized by control protein (β-actin) levels, and reported in a bar graph. One-way ANOVA: *P < 0.01 vs sham; ^##^P < 0.05 vs SCI.

In our previous paper [14] we reported that JNK MAPK activation is enhanced 24 h after SCI and that systemic treatment with CGS21680 prevents such activation. In the present paper, we confirm that a significant increase in phospho-JNK MAPK levels occurs 24 h after SCI (Figure 8). SCH58261, administered intraperitoneally three times in 24 hours, prevented SCI-induced JNK MAPK activation as evaluated by western blot (Figure 8).

### Effect of CGS21680 and SCH58261 centrally applied into spinal cord of SCI mice

**Figure 9:**
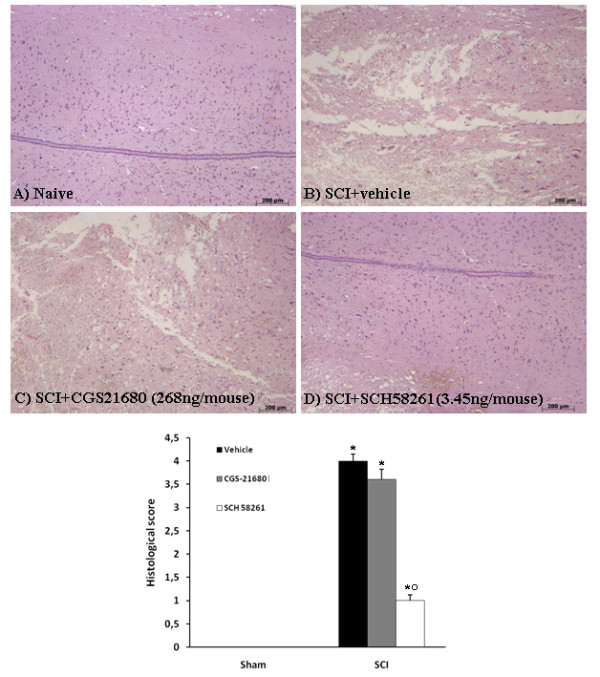
**Effect of SCH58261 and CGS21680 treatment on histological alterations when centrally applied after SCI**. Significant damage to the SC from SCI-operated mice in the perilesional area was assessed by the presence of edema as well as alteration of the white matter 24 h after injury (B). Notably, significant protection from SCI was observed in tissue collected from SCH58261-treated mice (D), whereas CGS21680, when locally applied on spinal cord tissue, did not protect against SCI (C). This figure is representative of at least 3 experiments performed on different experimental days. The histological score (d) was evaluated by an independent observer. ND: not detectable. *P < 0.01 vs sham; ^°^P < 0.01 vs SCI.

Since adenosine A_2A _receptor agonists and antagonists are protective against SCI when systemically administered, we applied these drugs directly into SC in order to understand their site of action. Twenty-four hours after SCI, significant damage to SC was observed in perilesional areas as assessed by alteration of the white matter when compared with sham-operated mice (Figure 9A, B). Notably, significant protection against SCI was observed in SCH58261-treated mice, in which SCH58261 was centrally applied (3.45 ng/mouse dissolved in 100 μl 10% DMSO) to the SC injury site 1 hour, 6 hours and 10 hours after SCI (Figure 9D). On the contrary, CGS21680, centrally applied (268 ng/mouse dissolved in 100 μl 10% DMSO) to the SC injury site 1 hour, 6 hours and 10 hours after SCI, did not protect against SCI (Figure 9C). SCH58261, applied at the higher dose of 35 ng/mouse, did not protect against SCI.

## Discussion

In the present paper we demonstrate that the adenosine A_2A _receptor antagonist SCH58261, systemically and continuously administered after SCI, protects from motor deficits up to 10 days after trauma. The A_2A _antagonist, systemically administered starting 1 hour after trauma, protects from tissue damage, demyelination, expression of death signals such as TNF-α, Fas-L, PAR, Bax; and from activation of JNK MAPK, while Bcl-2 expression is increased 24 hours later. Also when centrally applied, SCH58261 protects from tissue damage as evaluated 24 h after SCI. On the contrary, the selective adenosine A_2A _receptor agonist, CGS21680, centrally applied, is not protective.

In our previous study we showed that the selective adenosine A_2A _receptor agonist CGS21680, systemically administered after SCI, clearly reduces motor deficits for up to 19 days after SCI, and 24 hours after SCI protects against tissue damage and different inflammatory readouts [14]. On the basis of results that both adenosine A_2A _receptor agonists and antagonists, systemically administered after SCI with the same administration protocol, are protective against SCI, we considered the possibility that protective effects of A_2A _agonists could be due to A_2A _receptor desensitization at a spinal level. Here we demonstrate that after SCI, adenosine A_2A _receptor expression is definitely increased in damaged spinal cord as evaluated by western blot. Immunohistochemical analysis of SC in sham animals shows that adenosine A_2A _receptors are expressed by astrocytes and by a few microglial cells, are present on bundles of myelinated fibers, and are poorly expressed on neurons. After SCI, overexpression is clearly appreciated on neurons in agreement with results obtained after cross clamping of the infrarenal aorta [30]. Semiquantitative western blot analysis of spinal cord sections demonstrated that expression of A_2A _receptors is definitely reduced not only in CGS21680-treated mice but also in SCH58261-treated mice. This last result excludes the possibility that reduction of A_2A _receptors is due to the prolonged A_2A _agonist treatment, but likely indicates that reduction of A_2A _receptors occurs subsequent to protection induced by both A_2A _receptor agonist and antagonist.

The adenosine A_2A _receptor antagonist SCH58261, systemically and chronically administered after SCI, protects from motor deficits up to 10 days after trauma. After short-term systemic administration (1, 6 and 10 h after SCI), the A_2A _antagonist protected from tissue damage and inflammation and death signals such as TNF-α, Fas-L, PAR, Bax; while Bcl-2 expression was increased as evaluated at one time-point (24 h after SCI).

There are a number of mechanisms by which adenosine A_2A _receptors can play a role in central trauma and ischemia.

Adenosine A_2A _receptors are promoters of excitotoxicity by directly stimulating glutamate outflow, inhibiting glutamate uptake from neurons and glial cells and interacting with glutamate NMDA receptors [31]. It is well known that aspartate and glutamate play a critical role in the response of the CNS to ischemia/trauma [32,33]. After lumbar laminectomy, extracellular glutamate rapidly increases several fold after trauma in injured spinal tissue [34-36]. Much of the damage that occurs in the SC following traumatic injury is due to the secondary effects of glutamate excitotoxicity, Ca^2+ ^overload, and oxidative stress, three mechanisms that take part in a spiraling interactive cascade ending in neuronal dysfunction and death [37-39].

After lumbar laminectomy, it has been shown that adenosine also increases extracellularly soon after trauma [23]. The A_2A _receptor agonist, CGS21680, increases miniature excitatory postsynaptic currents in SC in the lamina IX neurones of spinal motoneurons, indicating that A_2A _receptors modulate excitatory synaptic transmission [40]. We have demonstrated that A_2A _antagonists reduce glutamate outflow in the first hours after brain ischemia [20].

The A_2A _antagonist SCH58261, when directly injected into the injured spinal cord at a concentration (3.45 ng/mouse) that can be reached in the SC after systemic administration, also protects from tissue damage as assessed 24 hours after SCI. This demonstrates that the protective effect of A_2A _antagonism is accounted for by antagonism of A_2A _receptors present on spinal neural cells. Our results coincide with those indicating that, when injected directly into the hippocampus, the A_2A _antagonist ZM241385 significantly reduces kainate-induced neuronal damage but the A_2A _agonist CGS21680 does not [41]. It is worth remembering that systemic administration of both CGS21680 and ZM241385 protects against hippocampal neuronal damage induced by intrahippocampal injection of the excitotoxin kainate [42].

We also observed that SCH58261 administered in SC at a higher concentration (35 ng/mouse) is no longer protective. It is interesting that SCH58261, systemically administered at dose of 0.01 mg/kg i.p. (the same dose utilized in the present study), protects against the glutamate increase induced by K^+ ^and kinolinic acid, but at the higher dose of 1 mg/kg i.p. is no longer protective [43,44]. These observations support the view that adenosine A_2A _receptor antagonist exerts its protective effects by reducing glutamate levels (and by inference, toxicity). Interestingly it was recently reported that the protective effects against behavioral deficit and against activation of different parameters of neuroinflammation, exerted by both A_2A _receptor agonists/antagonists systemically administered after brain traumatic injury, are dictated by local glutamate concentrations [45]. It is unlikely that the lack of protection by the higher SCH58261 concentration is due to a lack of selectivity for A_2A _receptors because in binding studies SCH58261 shows A_2A _receptor affinity in the low nM range (K_i _of 2.3 nM), lower A_1 _receptor affinity (K_i _of 121 nM) and no affinity for A_3 _receptors up to micromolar concentrations [46]. The effectiveness of A_2A _receptor antagonists seems to depend on a balance between beneficial effects at presynaptic sites, reducing glutamate outflow, and deleterious effect at postsynaptic sites increasing NMDA-induced toxicity [47]. A different degree of affinity of A_2A _antagonists for pre- and postsynaptic sites might help explain the finding that the neuroprotective effects are lost by increasing the concentration of SCH58261 [48].

The evidence favours the idea that A_2A _receptor antagonist administered at a lower concentration, by reducing glutamate outflow from neurons and glial cells of injured SC, reduces excitotoxicity. Since excitotoxicity drives an ensuing inflammatory cascade [25], reduction of excitotoxicity by the A_2A _receptor antagonist might well account for reduction of downstream effects consisting in production of inflammation and death signals such as TNF-α, Fas-L, PAR, and Bax; or increase of Bcl-2 expression after SC damage. Reduction of inflammation and death signals, in turn, might account for the persistent (up to 10 days) protection from motor deficit.

Although SCH58261 at a dose of 0.01 mg/kg is not active peripherally on heart rate or systemic blood pressure [49], and much evidence indicates that the protective effect of A_2A _antagonists is related to central local glutamate concentrations, it cannot be excluded that, when peripherally administered, part of the protective effects of A_2A _antagonists are mediated by peripheral cells. In this regard it is worth mentioning that inactivation of A_2A _receptors on BMDC attenuates ischemic brain injury [50] and brain trauma and also inhibits inflammatory cytokine production [45].

Not only are A_2A _antagonists protective, but there is robust evidence that adenosine A_2A _receptor agonists also protect against locomotor dysfunction and expression of death signals following SC ischemia-reperfusion and traumatic injury [11-15]. In attempting to shed light on the site of action accounting for the protective effects of A_2A _receptor agonists, we directly injected CGS21680 into injured SC at a concentration (268 ng/mouse) that can be reached in the SC after systemic administration.

In contrast to what was observed with the A_2A _receptor antagonist, the A_2A _agonist CGS21680 injected into injured SC was not protective against cell damage as assessed 24 hours after SCI. This demonstrates that the protective effect of the systemically administered drug is not attributable to activation of A_2A _receptors on central SC cells but rather is mediated peripherally. Li et al. [13] demonstrated that the protective effect from motor deficits of A_2A _agonists systemically administered after spinal trauma is lost in mice lacking A_2A _receptors on bone marrow-derived cells (BMDCs), but is restored in A_2A_-KO mice reconstituted with A_2A _receptors on BMDCs. This result identifies BMDCs as the targets of A_2A _agonists. Most studies have reported that selective activation of A_2A _receptors inhibits proinflammatory responses directly in BMDCs, including platelets, monocytes, some mast cells, neutrophils and T cells [51-53]. A_2A _and/or A_2B _receptors may be responsible for lymphocyte proliferation [54,55]. Consistent with its antiinflammatory and immunosuppressive role, the protective effects of adenosine A_2A _receptor stimulation have been observed in various models of autoimmune disease, such as rheumatoid arthritis [56], colitis [15,57], and hepatitis [58]. Therefore we must assume that the definite protection by A_2A _agonists systemically administered beginning 1 hour after SCI [11-15] is exerted at peripheral BMDCs resulting ultimately in reduced leucocyte infiltration and a reduced inflammatory cascade at the central level.

Twenty-four hours after SCI, clear signs of cell suffering are present, demonstrated by fragmented astrocytes having morphological features of damaged cells, by microglial cells that have the morphological features of activated cells and by bundles of myelinated fibers that appear disorganized and fragmented. The selective A_2A _adenosine receptor antagonist SCH58261 attenuated myelin damage in white matter as demonstrated by Luxol fast blue and by Weigert's and Oil red O coloration.

In agreement with our previous results [14], a significant increase in phospho-JNK MAPK levels was observed 24 h after SCI. Phospho-JNK MAPK was found *de novo *expressed in oligodendrocytes in the ventro-lateral portion of injured white matter but not in neurons, microglia or astrocytes [14]. The A_2A _receptor antagonist SCH58261 reduces JNK MAPK activation. Previous studies have demonstrated that the A_2A _adenosine agonists, systemically administered after SCI, also reduce JNK MAPK activation [14] and demyelination [13]. A reduction of JNK MAPK activation might account for better survival and/or functionality of mature myelinating oligodendrocytes as well as reduced damage to developing oligodendrocyte progenitors. In fact, previous work has demonstrated that activation of JNK MAPK is involved in oligodendrocyte death [59,60], and activation of JNK MAPK has been described in oligodendrocytes in multiple sclerosis lesions where oligodendrocytes are major targets of the disease [61]. Oligodendroglia are extremely sensitive to glutamate receptor overactivation and ensuing oxidative stress [62-64] as well as to cytokines and adenosine [65]. Glutamate toxicity in brain cortical cultured oligodendrocytes is reduced by the pan-JNK inhibitor SP600125 [66]. When considering the possibility that A_2A _receptors directly control JNK MAPK activation in oligodendrocytes, the only available evidence from studies of mouse macrophages shows that adenosine does not modify phosphorylation of JNK MAPK [67]. It is likely that activation of JNK MAPK after SCI is an epiphenomenon consequent to an inflammatory cascade that is driven by both excitotoxicity and infiltration. Therefore the A_2A _receptor antagonist, systemically administered, by reducing excitotoxicity and the ensuing inflammatory cascade can reduce JNK MAPK activation. The A_2A _receptor agonist, by reducing leucocyte infiltration and the ensuing inflammatory cascade at a central level, can also reduce JNK MAPK activation.

## Conclusions

Protection by A_2A _antagonist, systemically administered beginning 1 hour after SCI, is afforded centrally and is attributable to precocious antagonism of excessive glutamate transmission and of the ensuing inflammatory cascade.

Protective effects afforded by A_2A _agonist, systemically administered beginning 1 hour after SCI, are likely due to peripheral actions that may mediate inflammatory responses.

When attempting to use adenosine A_2A _active drugs to protect against SCI, attention should be given to the dose of antagonists to be used and to administration time after injury. It is likely that A_2A _antagonists, at low doses, provide protection by control of excessive excitotoxicity, while A_2A _agonists provide protection by controlling a massive infiltration in the hours after SCI. Results reported in the present work might be useful for envisaging novel strategies for control of acute SC injury and later secondary injury.

## Abbreviations

CGS21680: 2-[p-(2-carboxyethyl)-phenethylamino]-5'-N-ethylcarboxamidoadenosine; BMDCs: bone marrow-derived cells; JNKs: c-jun N-terminal kinases; H&E: hematoxylin/eosin; MAPKs: Mitogen-activated protein kinases; MPO: myeloperoxidase activity; PMN: polymorphonuclear leukocyte; SC: spinal cord; SCI: spinal cord injury; SCH58261: (7-(2-phenylethyl)-5-amino-2-(2-furyl)-pyrazole-[4,3-*e*]-1,2,4-triazolo[1,5-*c*] pyrimidine).

## Competing interests

The authors declare that they have no competing interests.

## Authors' contributions

IP participated in setting up the model of SCI and acquisition of data. AM, SC and FC participated in immunohistological studies, in the experimental design and in the acquisition of data. TM partecipated in the acquisition of data from Fluorescence Deconvolution Microscopy. EM and EE participated in setting up western blot assays and acquisition of data. PB, SC and FP provided the study concept, design and supervision. All authors provided analysis and interpretation and wrote the manuscript. All authors read and approved the final manuscript
